# Non-invasive ventilation (NIV) as an aid to rehabilitation in acute respiratory disease

**DOI:** 10.1186/1471-2466-11-58

**Published:** 2011-12-16

**Authors:** Fran Dyer, Lizzie Flude, Farid Bazari, Caroline Jolley, Catherine Englebretsen, Dilys Lai, Michael I Polkey, Nicholas S Hopkinson

**Affiliations:** 1 The NIHR Respiratory Biomedical Research Unit at Royal Brompton and Harefield NHS Foundation Trust and Imperial College, London SW3 6NP UK; 2 Chelsea and Westminster NHS Foundation Trust, 369, Fulham Rd, London SW10 9NH, UK

## Abstract

**Background:**

Non-invasive ventilation (NIV) can increase exercise tolerance, reduce exercise induced desaturation and improve the outcome of pulmonary rehabilitation in patients with chronic respiratory disease. It is not known whether it can be applied to increase exercise capacity in patients admitted with non-hypercapnic acute exacerbations of COPD (AECOPD). We investigated the acceptability and feasibility of using NIV for this purpose.

**Methods:**

On a single occasion, patients admitted with an acute exacerbation of chronic respiratory disease who were unable to cycle for five minutes at 20 watts attempted to cycle using NIV and their endurance time (T_lim_) was recorded. To determine feasibility of this approach in clinical practice patients admitted with AECOPD were screened for participation in a trial of regular NIV assisted rehabilitation during their hospital admission.

**Results:**

In 12 patients tested on a single occasion NIV increased T_lim _from 184(65) seconds to 331(229) seconds (p = 0.04) and patients desaturated less (median difference = 3.5%, p = 0.029). In the second study, 60 patients were admitted to hospital during a three month period of whom only 18(30)% were eligible to participate and of these patients, only four (7%) consented to participate.

**Conclusion:**

NIV improves exercise tolerance in patients with acute exacerbations of chronic respiratory disease but the applicability of this approach in routine clinical practice may be limited.

**Trial registration:**

http://www.controlled-trials.com/ISRCTN35692743

## Background

Exercise limitation is a cardinal feature of COPD which is particularly marked in patients who require hospital admission. It is directly related to skeletal muscle weakness [1-3] and quadriceps weakness is associated with increased mortality in COPD [4]. Patients with low physical activity levels are more likely to be admitted to hospital [5] and exacerbations themselves lead to a dramatic reduction in physical activity [6] and health status [7] which can be prolonged, reflected in reduced time spent outdoors [8]. Activity limitation is also associated with a greater likelihood of relapse after discharge following accident and emergency department attendance [9].

Although muscle weakness is multifactorial, disuse is likely to be the major reversible factor with loss of strength most pronounced in the muscles of locomotion [10]. In healthy older people 10 days bed rest produces a 20% fall in quadriceps strength [11]. An acute fall in strength has been noted in patients admitted with acute exacerbations of COPD (AECOPD) [12-14] and decline in fat free mass in COPD is associated with exacerbation frequency [15].

Pulmonary rehabilitation is a well established therapeutic strategy for out-patients with COPD, improving exercise capacity and quality of life as well as reducing hospital admissions and health care costs [16-19]. Outpatient pulmonary rehabilitation started within 10 days of discharge following AECOPD has been shown to improve exercise capacity, quality of life and readmission rate [20,21]. Benefit has also been seen in patients undergoing a supervised exercise program in their own homes. A six week program begun straight after discharge, with twice weekly visits, improved shuttle walk distance, quadriceps strength and quality of life compared to usual care with a trend towards fewer exacerbations at three months [22].

There are also some data looking at in-patient programs for patients with AECOPD after they have recovered sufficiently but before discharge [23,24]. In these studies patients were enrolled four to seven days after admission to hospital. The investigators found that walking distance almost doubled in the intervention arm whereas it did not change in the control group. The benefits of this early rehabilitation in terms of exercise capacity, breathlessness and quality of life were large and appeared to be maintained with unsupervised home exercise program suggesting that the benefits from preventing or reversing acute deteriorations of muscle strength are important and can be sustained relatively easily [24].

There are data to suggest that non-invasive ventilation (NIV) can be used to allow stable COPD patients with ventilatory limitation to exercise at higher intensities [25-33] and it has also been used to allow mobilisation in patients on an intensive care unit [34]. One study investigating the effect of NIV on exercise tolerance in acute hypercapnic exacerbations of respiratory disease [35] showed that NIV with oxygen entrained improved six minute walk distance, with reduced dyspnoea and improved SpO_2 _compared to oxygen alone. It is therefore possible that, as in stable disease, NIV could support rehabilitation during an acute episode, allowing patients to exercise who would otherwise be too breathless and so prevent loss of muscle strength and functional capacity.

We present the results of two studies. The aims of the studies were: 1) to determine the effect of NIV on exercise tolerance in patients admitted to a specialist respiratory hospital with an acute exacerbation of chronic respiratory disease on a single occasion and 2) to investigate the feasibility of enrolling patients with non-hypercapnic AECOPD into a trial of NIV assisted rehabilitation.

## Methods

The two arms of the study were carried out independently, Study 1 looking at the effect of NIV on a single occasion at Royal Brompton Hospital and Study 2, looking at the feasibility of regular NIV assisted exercise at Chelsea and Westminster Hospital. Ethical approval was granted by the South West London Ethics committee and written consent was given by all participants. The initial intention was to pursue a randomised controlled trial as registered at http://www.controlled-trials.com/ISRCTN35692743 but only the results of initial feasibility studies are presented here.

### Acute effect of NIV on exercise tolerance (Study 1)

Patients admitted to the Royal Brompton Hospital with an acute deterioration of chronic lung disease (including Cystic Fibrosis [CF], Bronchiectasis), who were judged by the clinical physiotherapy team to have significantly impaired exercise capacity and were not expected to be able to cycle for five minutes, were invited to participate. Patients were naive to NIV-assisted exercise but nocturnal NIV use was not an exclusion. Patients had to be able to follow instructions and be able to use an exercise bike. Patients were excluded if they were known to be acidotic (pH < 7.35), febrile, or had a heart rate (HR) > 120 min^-1^, a systolic blood pressure < 100 mmHg or were considered unsafe to exercise by the medical team.

#### Unassisted exercise

Patients were asked to cycle on an upright static bike (Horizon Fitness BSC 400) at 20W at 40-60 RPM for up to five minutes without ventilatory support. Oxygen was entrained at the baseline level predetermined by the clinical team responsible for the patient by assessing oxygen requirements during mobilisation. This was not adjusted during exercise. Patients received standardised encouragement to try to continue for five minutes. HR, O_2 _saturation and symptoms (Borg score for breathlessness and leg discomfort) were recorded. If patients felt unable to cycle for five minutes (symptom limited) or suffered significant desaturation (stopped by supervising physiotherapist if they desaturated by more than 4% from baseline and to less than 88%) whilst cycling they went on to try NIV assisted exercise.

#### NIV assisted exercise

NIV was delivered using a NIPPY ST+ via a full face mask in NIV naïve individuals. If patients were already using NIV at night their own machine and interface was used. If patients were unable to tolerate a full face mask a mouthpiece was trialled. For those naive to NIV a pressure support of 10 cmH_2_O was used as a baseline with a PEEP of 5 cmH_2_O. Ventilator settings were adjusted as necessary to allow for increased ventilatory demands and to maximise patient comfort during exercise. Oxygen was entrained at the baseline flow rate and not adjusted during exercise. Patients then cycled for up to 20 minutes on a static bike at 20 watts. For a small number of patients workload was increased during NIV assisted exercise as they found the low resistance uncomfortable. This meant that for some individuals a change in Tlim could be reported and in others a change in total work done (watts × time).

#### Outcome measures

Total time cycled was recorded and SpO_2 _was monitored throughout and recorded at one minute intervals. Breathlessness and leg fatigue were measured using the Borg score immediately post exercise. Participants were asked to rate the acceptability of exercising with NIV using a visual analogue scale scored (0-10). Patient remarks about the difference in ability to exercise were also recorded.

### Feasibility of NIV assisted rehabilitation (Study 2)

Patients admitted to Chelsea and Westminster hospital with an AECOPD as per current UK guidelines [36] were screened for eligibility for a trial to evaluate regular NIV-assisted exercise while they were an inpatient. All patients were treated with antibiotics and oral corticosteroids. To be included, patients had to be expected to remain an in-patient for more than 24 hours, be expected to survive the admission and be safe to exercise according to the criteria in the first part of the study. If patients failed the latter criteria (heart rate, blood pressure, fever or acidosis) they could be reassessed on subsequent days. Where patients were eligible the study was proposed to them by a member of the clinical team and they were invited to participate. No specific definition of exercise limitation was used as an inclusion criterion, as this study was designed with the knowledge that patients admitted to hospital with an AECOPD become weaker whilst in hospital and because patients who were not exercise-limited would be likely to be managed as outpatients [12-14].

### Statistical analysis

Data were analysed using SPSS. Descriptive data for continuous variables with a normal distribution are presented as mean and standard deviation (SD) or median and range for data not normally distributed. The paired t test or the Wilcoxon signed rank test for non-parametric data and the chi squared test for categorical data were used to identify any differences whilst exercising with and without NIV. A significance level of < 0.05 was used for all comparisons.

## Results

### Effect of NIV on exercise tolerance (Study 1)

**Figure 1:**
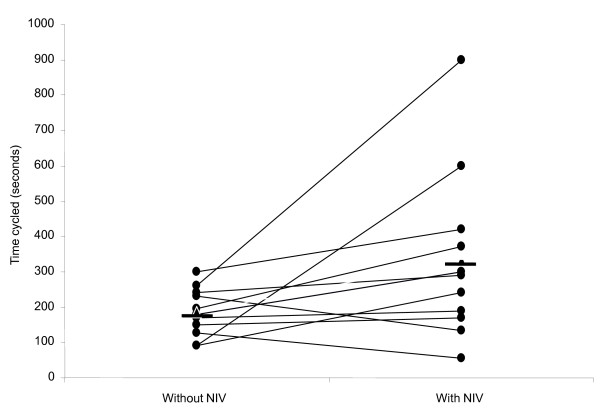
**Cycle endurance time at 20w increased with NIV support (p 0.04)**. n = 12 Horizontal bars represent mean values.

**Figure 2:**
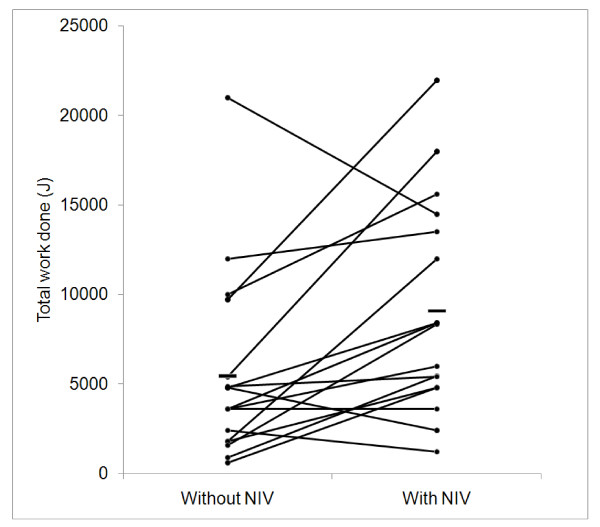
**Work done during cycle ergometry increased with NIV support (p = 0.016)**. Horizontal bar represents mean values.

Twenty-five patients were studied (Table 1). Eleven (44%) patients had cystic fibrosis, 12 (48%) bronchiectasis, one (4%) had primary ciliary dyskinesia and one (4%) had obesity hypoventilation and dilated cardiomyopathy. Forty-three per cent had had prior exposure to NIV. Eight patients were able to cycle for five minutes at 20W and therefore did not proceed to NIV-assisted exercise. In 12 patients exercising with a fixed workload, T_lim _increased from 184(65) sec to 331(229) sec (p = 0.04) (Figure 1) (table 2). In five patients, workload was increased whilst exercising on NIV so only total work done is reported. In this subgroup median increase in exercise time was 85 secs (30%) and median total work done increased from 1560 (600-9720)J to 8336 (4800-21960)J (p = 0.043). 10 patients used supplemental oxygen at rest and during exercise (oxygen saturation values in tables 1 and 2 reflect this). In all patients (n = 17) work done increased from 5437 (600-21000)J to 9081 (1200-21960)J (p = 0.016) (Figure 2).

**Table 1:** Patient characteristics for study 1

	All patients	Able to cycle > 5 minutes at 20W unassisted	Unable to cycle > 5 minutes at 20W unassisted	p
n (%male)	25 (58%)	8(88%)	17(44%)	0.08^‡^
Age (yrs)	48.0 (18.8)	42.9 (12.2)	50.6 (21.3)	0.35*
FEV_1 _(L)	0.84(0.49-2.85)	0.97 (0.68-2.85)	0.69 (0.49-1.40)	0.03^†^
FVC(L)	1.74 (0.80-4.55)	1.89 (1.06-4.55)	1.54 (0.80-2.31)	0.13^†^
Resting SpO_2_	94.9 (3.0)	96.0 (2.7)	94.4 (3.1)	0.21*
Resting HR	97.5 (15.1)	96.8 (12.1)	97.8 (16.3)	0.87*

Data are presented as mean (SD) or median (range)

* unpaired t test, ^† ^Wilcoxon signed rank test, ^‡ ^Chi squared

**Table 2:** Exercise parameters where workload held constant (Study 1)

	Without NIV	With NIV	P value
T_lim_	184.4 (65.2)	331.1 (229.1)	0.04*
Resting SpO_2_	94.83 (2.17)	94.83 (2.55)	1.00*
Resting HR	94.33 (16.99)	93.83 (18.45)	0.86*
ΔSpO_2 _(%)	-7.33 (5.12)	-3.83 (4.90)	0.029^†^
ΔHR (min^-1^)	16.33 (11.54)	16.33 (7.46)	0.93^†^
Borg dyspnoea	3.72 (1.90)	3.86 (1.87)	0.62^†^
Borg Leg discomfort	11.36 (2.29)	11.00 (2.86)	0.83^†^

All data presented as mean (SD) or median (range). Tlim, endurance time on cycle ergometer at 20W; SpO_2_, oxygen saturation; HR, heart rate. Borg scores were recorded at end of exercise. *paired t test, ^†^Wilcoxon signed rank test.

Change in total work done did not differ significantly between those naïve to NIV and those using nocturnal NIV with a mean change of 3263 (6028)J vs 4262 (3956)J respectively (p = 0.7).

It took an estimated 15 minutes for most patients to become familiarised with the mask and machine and to optimise their ventilator settings at rest. Patients were thus able to cycle with NIV with only a short introduction to the equipment. Patients comments were recorded and were both positive and negative including 'claustrophobic' and 'suffocating' as well as 'pleasant sensation', 'felt mask made exercise easier, after cycling for a while I appreciated having the oxygen' and 'my legs didn't hurt so much'. However, all patients who exercised with NIV stated they would be prepared to repeat the intervention even though some (35%) scored five or more on the visual analogue scale for discomfort with a mean score of 4.3 (3.3) range 0-10. No adverse events associated with exercise occurred.

### Feasibility of NIV assisted rehabilitation (Study 2)

Sixty patients admitted to Chelsea and Westminster hospital between February and May 2011 with an acute infective exacerbation of COPD were assessed for possible participation. Thirty-nine (65%) were male, mean age was 72 (12) years. Median length of stay (LOS) was 3 (0 to 19) days with 32% patients being discharged within 48 hours. Table 3 shows outcome of screening for eligibility.

**Table 3:** Screening for eligibility for study 2

	n	%
Met inclusion criteria	18	30%
Declined	10	56% of subtotal
Recruited	4	22% of subtotal
Other	4	22% of subtotal
Did not meet inclusion criteria	42	70%
Not fit to exercise	16	38% of subtotal
Poor prognosis	3	7% of subtotal
Expected stay < 24 hours	19	45% of subtotal
Other	4	10% of subtotal

Forty-two (70%) patients did not meet the eligibility criteria, the most common reasons being that they were only expected to have a short admission or were too unwell to exercise. Of those not fit to exercise, nine patients (56%) had a musculoskeletal problem including severe osteoarthritis of the hip, rheumatoid arthritis and a new Colles' fracture. Other reasons included dementia (13%) or neurological problems (13%). Of those considered eligible the majority 10/18 (56%) declined to participate and four were excluded for logistical reasons. Of those declining two patients did not wish to take part in research but most (70%) either did not feel they could exercise or did not want to exercise.

Of the four patients who agreed to participate it became apparent during the assessment process that one was too frail to continue (physiotherapist's assessment). One patient withdrew after the first exercise session as he found exercise too distressing due to severe breathlessness. Two patients continued NIV assisted exercise to discharge from acute care, however, of these one received only one exercise session and the other only two, due to short length of stays.

## Discussion

The main finding of study 1 was that non-invasive ventilation can significantly increase exercise tolerance in patients with acute exacerbations of chronic respiratory disease, reducing exercise desaturation. Exercise with NIV was practical, safe and well tolerated without significant adverse events. The finding of study 2 however, was that most patients admitted to hospital with non-hypercapnic AECOPD are not likely to be eligible for an NIV-assisted exercise intervention because their admission is too short, because they are too frail or have other co morbidities or because they decline to participate.

### Efficacy of NIV assisted exercise

In study 1 we showed that patients unable to exercise for five minutes at a low workload (20W) improved their exercise performance when exercising with NIV. As the tests were sequential and unblinded it is possible that there was a learning effect or a placebo effect. The fact that patients exercised for longer but to the same level of reported symptoms supports the view that NIV produced a genuine reduction in the work of breathing consistent with previous reports [25-33]. A more complex study design with patients exercising in random order could have been adopted, however we wished in particular in this pilot work to assess the feasibility of NIV in the acute setting, establishing whether it was possible to deliver this type of exercise intervention in this group of patients at all. We adopted a sequential approach, similar to that which might be adopted in clinical practice - if patients could cycle at 20W unassisted for five minutes then support would not be needed. An arbitrary time of five minutes cycling at 20 watts was used to define poor exercise capacity. We wished to investigate the effect of NIV assisted exercise in those with very limited exercise capacity as it is this group in whom additional support for exercise is likely to be most beneficial and thus where clinical resources would be best concentrated.

Where NIV was employed patients were able to adapt to using it quickly. A relatively low level of pressure support (10 cmH_2_O) was sufficient to improve exercise capacity consistent with previous work showing that NIV with a mean inspiratory pressure of 11 cmH_2_O was sufficient to improve walking distance in patients recovering from acute on chronic respiratory failure [35]. It remains to be established what the best strategy for augmenting exercise in this way is and we used two approaches - maintaining a fixed workload to see if endurance time increased or increasing the workload to give a greater training stimulus. Both approaches demonstrated that performance could be increased with the assistance of NIV. It should be noted that 3 patients displayed a reduction in the work done during exercise while using NIV and the possibility that NIV might impede rather than assist exercise in a given individual needs to be considered. The performance characteristics of the ventilator including trigger sensitivity, pressurisation rate and maximum flow capacity are important issues to be considered when using NIV to augment exercise as supporting ventilation in this context may be more demanding than during sleep.

### Feasibility of NIV assisted exercise during AECOPD

Study 1 was undertaken in patients admitted acutely to a specialist respiratory hospital who were a younger population with a range of chronic lung diseases and fewer co-morbidities than the AECOPD group in study 2. Thus in study 2 only 30% of those admitted with an acute exacerbation of COPD were suitable to approach to discuss participation. The main reason for exclusion was that 40% of patients were discharged from hospital within 48 hours of admission reflecting a general reduction in length of stay [37] in the UK and increased evidence for the effects of early supported discharge and similar schemes [38]. A shorter length of stay, though welcome, may have implications for assessing the impact of interventions on patients admitted with AECOPD, as the duration of treatment may make it difficult to establish whether there is an effect. This intervention may be more appropriate for patients who remain an in-patient for a longer period of time and future research studies should consider this. We selected patients who did not have a conventional clinical indication for NIV. Menadue *et al *recruited patients who were using NIV during an acute admission with hypercapnic respiratory failure due to AECOPD [35]. They found that NIV could increase exercise capacity which suggests that exercise with NIV in this patient population should be considered as an early strategy rather than waiting for patients to no longer require NIV before this is begun.

The low proportion of patients suitable for NIV assisted exercise has implications for trial design for studies with AECOPD with the low proportion of patients actually eligible factored into power calculations and the number of sites and duration of study required. For routine clinical practice, although NIV clearly can increase exercise capacity even in acutely breathless patients, many patients will decline to take part because they do not believe that exercise is possible or desirable when they are acutely unwell.

The main reason for declining to participate in the study was that patients did not want to or believed that they could not exercise. The correct balance between "rest" and "mobilisation" in acute illness needs to be defined and accepted by patients. This could have implications in clinical practice as some patients may choose not to participate in post exacerbation rehabilitation programmes with or without NIV. Man *et al *found that approximately 40% of patients assessed for eligibility for post-discharge pulmonary rehabilitation were not randomised [20]. Additionally this study required the patient to use an exercise bike. This has the advantage of stability but may be unfamiliar to some individuals. A different exercise strategy such as walking with the NIV on a wheeled walker or treadmill walking might have been more acceptable to older patients [35,39].

A limitation of the study is that spirometry data were not collected systematically for all patients admitted with AECOPD to assess this aspect of disease severity although the degree of exercise limitation caused by the respiratory disease exacerbation is of course the main issue and FEV_1 _is poorly predictive of exercise capacity in COPD.

At present, pragmatic use of NIV to enhance mobilisation in patients with a clinical indication for it, in particular those who are weaning from ventilatory support seems justified. If a more robust evidence base for the effectiveness of NIV to augment exercise in AECOPD is established then the case for its use could be made to patients more strongly, which may increase compliance beyond the levels observed in the present study.

## Conclusion

Non-invasive ventilation can improve exercise tolerance, with less desaturation in patients admitted to hospital with an exacerbation of chronic respiratory disease. Our experience suggests that it may be hard to recruit to studies in older populations with AECOPD.

## Competing interests

The authors declare that they have no competing interests.

## Authors' contributions

NSH, LF, MIP, FB, CJ, FD, CE conceived the study, FD, FB, CJ performed the measurements, FD produced the first draft of the paper which other authors contributed to and all approved the final version.

## Pre-publication history

The pre-publication history for this paper can be accessed here:

http://www.biomedcentral.com/1471-2466/11/58/prepub
